# Dysbiotic but nonpathogenic shift in the fecal mycobiota of patients with rheumatoid arthritis

**DOI:** 10.1080/19490976.2022.2149020

**Published:** 2022-12-06

**Authors:** Eun Ha Lee, Hyun Kim, Jung Hee Koh, Kwang Hyun Cha, Kiseok Keith Lee, Wan-Uk Kim, Cheol-Ho Pan, Yong-Hwan Lee

**Affiliations:** a Natural Product Informatics Research Center, KIST Gangneung Institute of Natural Products, Gangneung, Korea; b Interdisciplinary Program in Agricultural Genomics, Seoul National University, Seoul, Korea; c Department of Agricultural Biotechnology, Seoul National University, Seoul, Korea; d Division of Rheumatology, Department of Internal Medicine, College of Medicine, the Catholic University of Korea, Seoul, Korea; e Center for Integrative Rheumatoid Transcriptomics and Dynamics, College of Medicine, the Catholic University of Korea, Seoul, Republic of Korea; f Center for Plant Microbiome Research, Seoul National University, Seoul, Korea; g Plant Immunity Research Center, Seoul National University, Seoul, Korea; h Research Institute of Agriculture and Life Sciences, Seoul National University, Seoul, Korea

**Keywords:** Fecal microbiota, *Candida*, *Aspergillus*, dysbiosis, rheumatoid arthritis

## Abstract

Rheumatoid arthritis (RA) is closely associated with the oral and gut microbiomes. Fungal cell wall components initiate inflammatory arthritis in mouse models. However, little is known regarding the role of the fungal community in the pathogenesis of RA. To evaluate the association between RA and the gut microbiome, investigations of bacterial and fungal communities in patients with RA are necessary. Therefore, we investigated the compositions and associations of fecal bacterial and fungal communities in 30 healthy controls and 99 patients with RA. The relative abundances of *Bifidobacterium* and *Blautia* decreased, whereas the relative abundance of *Streptococcus* increased, in patients with RA. The relative abundance of *Candida* in the fecal fungal community was higher in patients with RA than in healthy controls, while the relative abundance of *Aspergillus* was higher in healthy controls than in patients with RA. *Candida* species-specific gene amplification showed that *C. albicans* was the most abundant species of *Candida*. Ordination analysis and random forest classification models supported the findings of structural changes in bacterial and fungal communities. *Aspergillus* was the core fecal fungal genus in healthy controls, although *Saccharomyces* spp. are typically predominant in Western cohorts. In addition, bacterial–fungal association analyses showed that the hub node had shifted from fungi to bacteria in patients with RA. The finding of fungal dysbiosis in patients with RA suggests that fungi play critical roles in the fecal microbial communities and pathogenesis of RA.

## Introduction

Rheumatoid arthritis (RA) is an autoimmune disease that mainly affects the synovium in joints. Synovial thickening leads to the destruction of joint cartilage and bone.^1–3^ Subsequently, RA can worsen and affect other joints, thereby increasing the risks of osteoporosis, Sjögren syndrome, heart diseases, and lung diseases.^4–7^ Although the pathogenesis of RA is incompletely understood, interactions among genetic, environmental, and lifestyle factors have been proposed. A significant genetic risk factor for RA is *HLA-DRB1*.^8^ Genome-wide association studies have shown that *PTN22, PADI4, STAT4*, and *TRAF1-C5* are associated with the onset of RA.^9^ Notably, the HLA-DRB1*0405 allele is closely associated with RA severity and susceptibility in Koreans.^10,11^ Clinical and experimental animal studies have shown that infection with *Porphyromonas gingivalis, Proteus mirabilis*, Epstein–Barr virus, or mycoplasma contributes to RA pathogenesis.^12^ The involvement of microbes in the etiopathogenesis of RA has prompted the investigation of relationships between RA and changes in human-associated microbial communities.

Dysbiosis has been identified in the fecal bacterial communities of patients with RA. Generally, gut microbial diversity is lower in patients with RA than in healthy individuals.^13^ Differences in the bacterial abundance were also observed. Specifically, the abundances of *Prevotella copri, Collinsella, Eggerthella*, and *Lactobacillus* increased in patients with RA, while the abundances of *Bacteroides, Faecalibacterium, Veillonella*, and *Haemophilus* decreased in those patients.^14,15^ Among the bacteria affected by RA, *P. copri* is predominantly present in the feces of patients with early RA; this species has been implicated in RA pathogenesis.^16^ Treatment for RA also affects the composition of the gut bacterial community. For example, etanercept increased the abundances of the *Cyanobacteria* and *Nostocophycideae* classes and the *Nostocales* order; it decreased the abundances of the *Deltaproteobacteria* class and the *Clostridiaceae* family.^17^ Patients who received methotrexate (MTX) showed a reduced abundance of *Enterobacteriales* and partial community restoration, compared with the typical dysbiotic community in patients with RA.^13,17,18^ Additionally, the bacterial community is affected by the presence of rheumatoid factor (RF) or anti-citrullinated protein antibody (ACPA), which are markers used to classify RA. Moreover, the C-reactive protein level (CRP) and erythrocyte sedimentation rate (ESR) are associated with gut microbiome dysbiosis in patients with RA.^17,19,20^

Similar to studies of bacteria, an association between fungi and RA pathogenesis has been reported. Intraperitoneal injections of a fungal cell wall component (zymosan or fungal β-glucan) into SKG mice in a specific pathogen-free laboratory resulted in the induction of autoimmune arthritis, whereas injections of an antifungal agent and antifungal cell wall component did not.^21^ Therefore, fungi are essential for the initiation of autoimmune arthritis. In a previous study that investigated the gut fungal community of patients with RA in China, the abundance of *Pholiota, Scedosporium*, and *Trichosporon* were lower than in healthy controls. *Suhomyces* and *Trebouxia*, two fungal genera abundant in patients with RA, were positively correlated with RA biomarkers.^22^ However, the effects of the fecal fungal community on RA have been less extensively investigated than the effects of the bacterial community.

Here, we investigated the fecal bacterial and fungal communities of patients with RA. We aimed to i) evaluate the fecal bacterial and fungal compositions and their interkingdom associations, ii) identify key taxa or operational taxonomic units (OTU) associated with compositional shifts in the fecal bacterial and fungal communities, and iii) examine the effects of medications on the fecal fungal community. The abundance of *Candida* was increased, while the abundance of *Aspergillus* was decreased, in the feces of patients with RA. The abundances of *Candida* and *Aspergillus* showed contrasting correlations with clinical factors used for RA diagnosis. In addition, the hub node, which plays a central role in bacterial–fungal associations, shifted from fungi to bacteria in patients with RA. Finally, the abundance of *Candida albicans* was affected by treatment for RA. Our study provides insight into the crucial roles of the fungal community in pathogenesis of RA.

## Results

### Descriptive statistics

The demographic and clinical features of patients with RA (RA) and healthy controls (HC) are shown in Table 1. The study included 99 RA and 30 HC. Ninety-one samples (91.9%) in the RA group were from women, while 100% of samples in the HC group were from women. The mean participant ages were 57.8 ± 10.1 years in the RA group and 46.9 ± 3.5 years in the HC group. RF and ACPA positivity were detected in 77 (77/93, 82.8%) and 75 (75/98, 76.5%) patients, respectively. Of the RA patients, 87 (87.9%) were prescribed conventional synthetic disease-modifying antirheumatic drugs (csDMARDs), while 40 (40.4%) were prescribed biologics (including 32 patients who were prescribed both biologics and csDMARDs). Medications used by patients with RA are listed in Table S1.

**Table 1.** Characteristics of study participants.

Characteristics	Healthy controls (*n* = 30)	Patients with RA (*n* = 99)
**Demographics**		
Mean age (year)	46.9 ± 3.5	57.8 ± 10.1
Female proportion	30 (100%)	91 (91.9%)
BMI	23.9 ± 3.0	22.8 ± 2.7
**Disease characteristics**		
RF positivity at entry of study	2 (6.7%)	77 (n = 93, 82.8%)
Anti-CCP positivity at entry of study	NA	75 (n = 98, 76.5%)
Disease duration, median (IQR), year	NA	8.9 (0.1–40)
CRP, median (IQR), mg/dL	NA	0.5 (0.0–5.1)
ESR, median (IQR), mm/hr	NA	13.3 (2.0–70.0)

### Fecal microbial community composition

**Figure 1.**
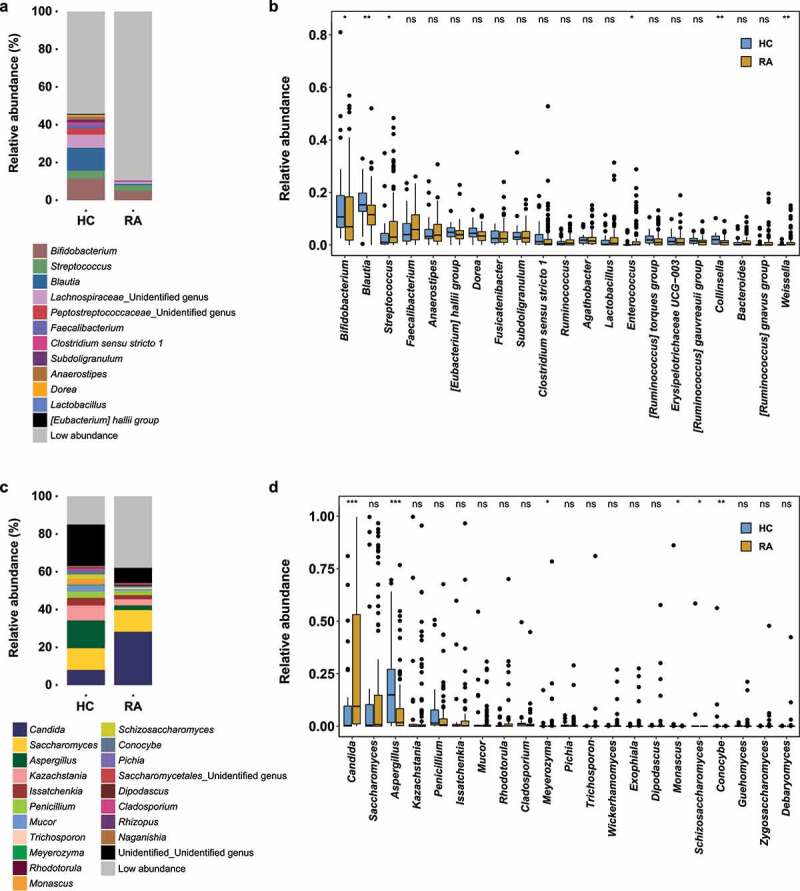
Fecal microbial community composition in healthy controls and patients with RA. (a) Bacterial community composition at the genus level. (b) Pairwise comparison of abundant bacterial genera. (c) Fungal community composition at the genus level. (d) Pairwise comparison of abundant fungal genera. Taxa with abundance < 0.5% are grouped as “Low abundance.” In panels b and d, boxes and lines represent the interquartile ranges (Q3-Q1) and medians of relative abundances, respectively. Black dots indicate potential outliers. Lower and upper whiskers show minimum and maximum relative abundances of genera. Statistical significance was estimated by two-sided Mann–Whitney U test. ***, *P* < .001; **, *P* < .01; *, *P* < .05; ns, *P* > .05 (not significant). HC, healthy controls; RA, patients with RA.

Distinct compositional differences between HC and RA were observed in the fecal bacterial community (Figure S1). *Bifidobacterium, Streptococcus, Blautia, Lachnospiraceae*, and an unidentified species were abundant in both the RA and HC groups (Figure 1a). However, genera with relative abundances < 0.3% constituted 89.4% of genera in the RA group and 54.2% of genera in the HC group (Figure 1a). Furthermore, the abundances of *Bifidobacterium* and *Blautia* were higher in the HC group than in the RA group; the abundance of *Streptococcus* was higher in the RA group than in the HC group. These differences were statistically significant (*Bifidobacterium, P* = .0299; *Blautia, P* = .0024; *Streptococcus, P* = .0195) (Figure 1b).

The most abundant fungal phyla were *Ascomycota, Basidiomycota*, and *Mucoromycota* (Figure S2). The ratio of *Basidiomycota* to *Ascomycota* was greater in the HC group than in the RA group (5.28%:65.7% in the HC group; 4.55%:75.42% in the RA group), while the proportion of *Mucoromycota* was greater in the HC group than in the RA group (mean relative abundances: 4.35% in the HC group and 1.74% in the RA group). The abundance of *Saccharomycetes* was greater in the RA group (HC, 35.2%; RA, 59.9%), while the abundance of *Aspergillaceae* was greater in the HC group (HC, 23.9%; RA, 10.0%) (Figure S2).

At the genus level, *Candida, Saccharomyces*, and *Aspergillus* were the most abundant fungi (Figure 1c, d). The relative abundance of *Candida* (*P* = .00013) was significantly greater in the RA group than in the HC group, while the relative abundance of *Aspergillus* (*P* = .00092) was significantly greater in the HC group than in the RA group (Figure 1d). Furthermore, the abundances of the genera *Kazachctania, Issatchenkia, Penicillium*, and *Mucor* tended to be greater in the RA group, although these findings were not statistically significant (Figure 1c, d). In contrast to the findings in a Western cohort, the abundance of *Saccharomyces* did not differ between the two groups (Figure 1d; mean relative abundances: HC, 12.9%; RA, 14.2%; *P* = .6204). Therefore, RA may be associated with compositional shifts in the fecal bacterial and fungal communities.

### Fecal microbial diversity

**Figure 2.**
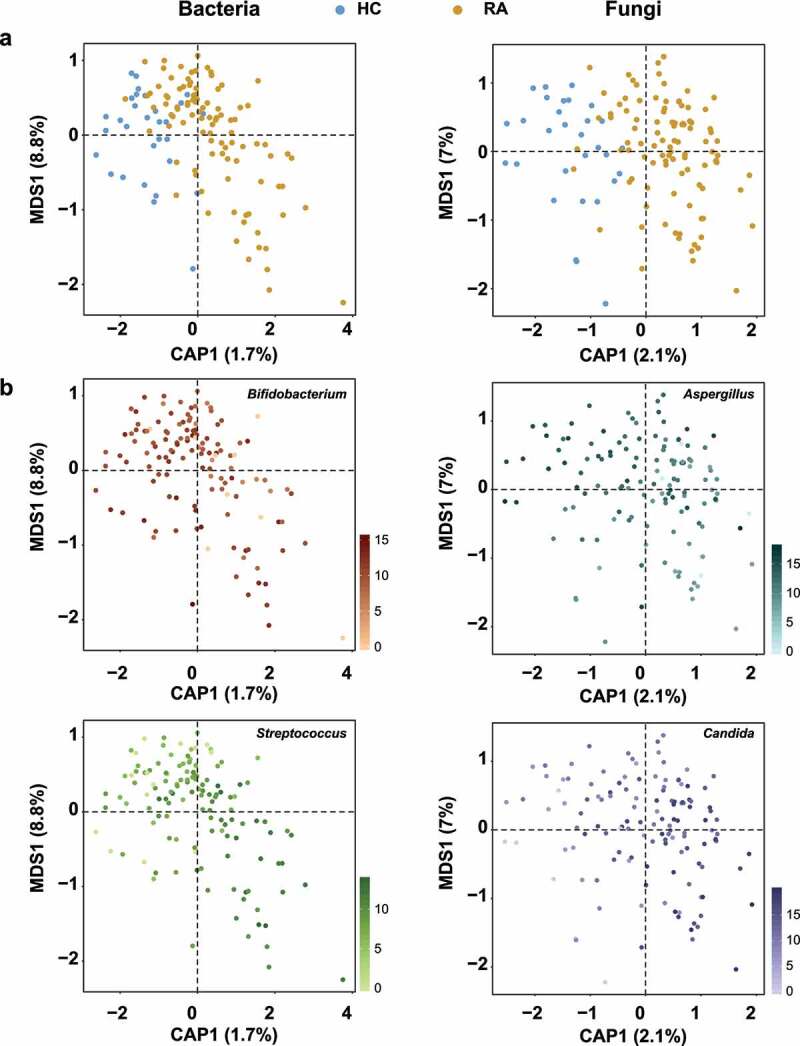
Ordination analysis of fecal bacterial and fungal communities in healthy controls and patients with RA. Compositional variations among samples were estimated by CAP, based on the Bray–Curtis distance metric. (a) Changes in composition of fecal bacterial and fungal communities. HC samples are shown in blue; RA samples are shown in dark yellow. (b) Ordination analysis according to the relative abundances of abundant genera. Greater intensity denotes higher relative abundance. Left and right sides of panels a and b are bacterial and fungal communities, respectively.

We next investigated the effects of RA on microbial community diversity. The alpha diversity indices, including OTUs, Shannon, and Simpson indices, of bacteria and fungi did not significantly differ between groups (all *P* > .05) (Figure S3). In Canonical analysis of principal coordinates (CAP), the constrained ordination analysis showed that bacterial and fungal communities were clearly separated into HC and RA groups (Figure 2a), although the unconstrained principal coordinates analysis did not show clear clustering of microbial communities according to RA status (Figure S4). Permutational analysis of variance (PERMANOVA) indicated significant compositional differences in the bacterial (R^2^ = 0.01746, *P* = .0002) and fungal communities (R^2^ = 0.0216, *P* = .0001) of the RA group (Table S2). The relative abundances of *Bifidobacterium, Streptococcus, Aspergillus*, and *Candida* differed between the two groups (Figure 2b). Although RA did not affect the richness or diversity of fecal microbial communities, it significantly affected beta diversity by shifting the taxonomic compositions of the fecal bacterial and fungal communities.

### RA patient-associated bacterial and fungal OTUs

We investigated the relative abundances of OTUs in the HC and RA groups by linear discriminant analysis effect size (LEfSe) analysis. Among 1338 bacterial OTUs and 1595 fungal OTUs, 57 bacterial OTUs and 45 fungal OTUs were differentially abundant (Figures S5a, S6a). In total, 14 bacterial OTUs and 10 fungal OTUs were more abundant in the RA group than in the HC group. The RA-enriched OTUs belonged to the fungal genera *Candida, Meyerozyma, Penicillium, Aurobasidium, Xeromyces, Coprinopsis*, and *Wallemia*. Furthermore, 43 bacterial OTUs and 35 fungal OTUs were more abundant in the HC group than in the RA group. The HC-enriched OTUs belonged to the fungal genera *Aspergillus, Conocybe, Monascus*, and *Schizosaccharomyces*.

**Figure 3.**
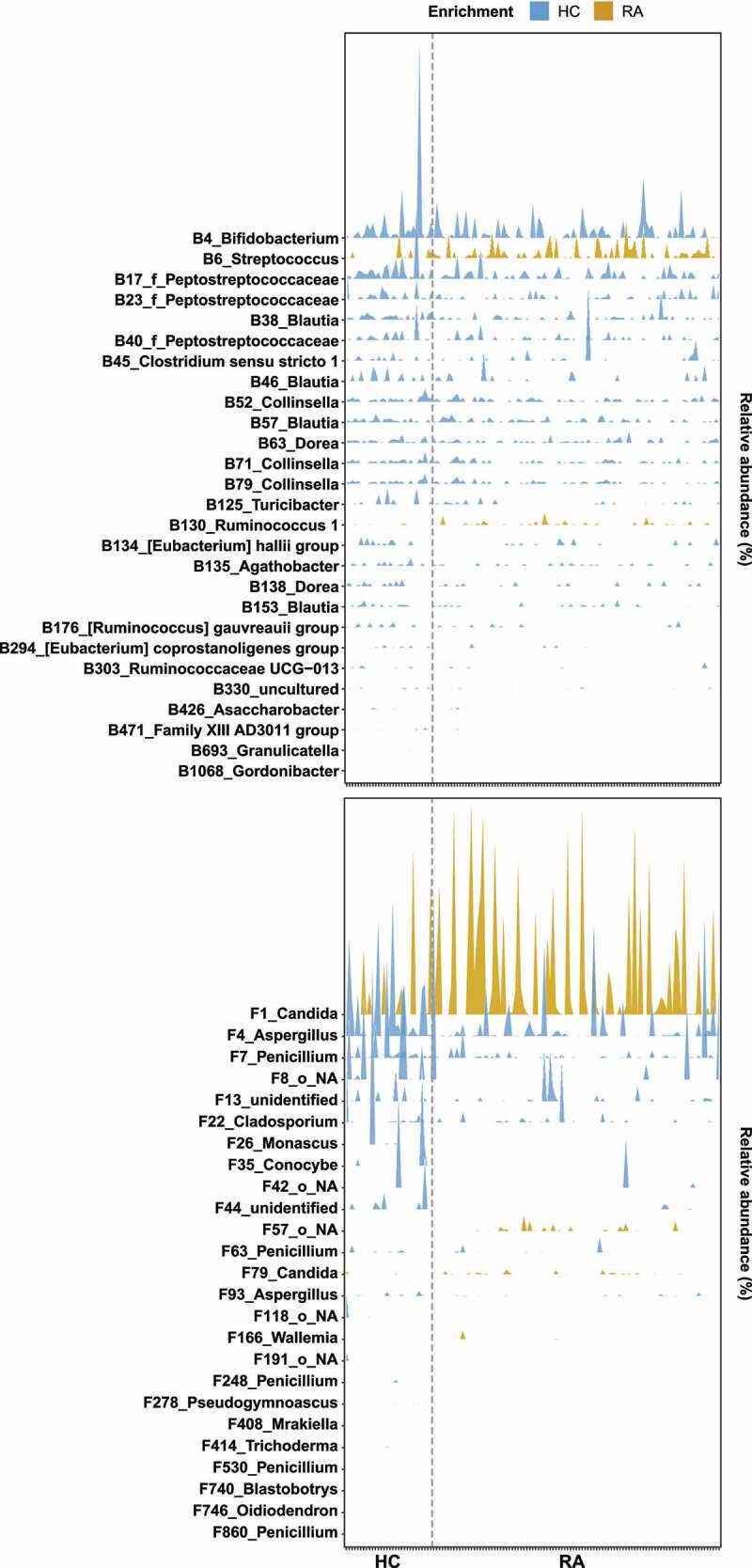
Microbial signatures associated with RA. A random forest model was used to identify OTUs that explain the gut bacterial (a) and fungal (b) communities. OTUs are colored based on their classification as “HC-enriched” and “RA-enriched,” based on the results of differential abundance analysis (Figure S5a, S6a). Random forest models were constructed using a 10-fold cross-validation method. OTUs are arranged along the y-axis according to total abundance. Each mark on the x-axis indicates an individual HC or RA sample.

We investigated RA-associated OTUs via machine learning-based classification. For this analysis, we constructed random forest classification models for bacterial and fungal communities. The random forest models revealed that 70 bacterial OTUs and 70 fungal OTUs were needed to classify HC and RA samples (Figures S5b, S6b). Among these OTUs, 27 bacterial OTUs and 25 fungal OTUs were also identified by LEfSe (Figures 3, S5a, S6a). Two bacterial OTUs and four fungal OTUs were more abundant in the RA group, while the remaining OTUs were more abundant in the HC group (Figure 3). These findings imply that decreased abundances of bacterial and fungal OTUs contributed to compositional differences between the HC and RA groups.

### Fecal bacterial–fungal associations

**Figure 4.**
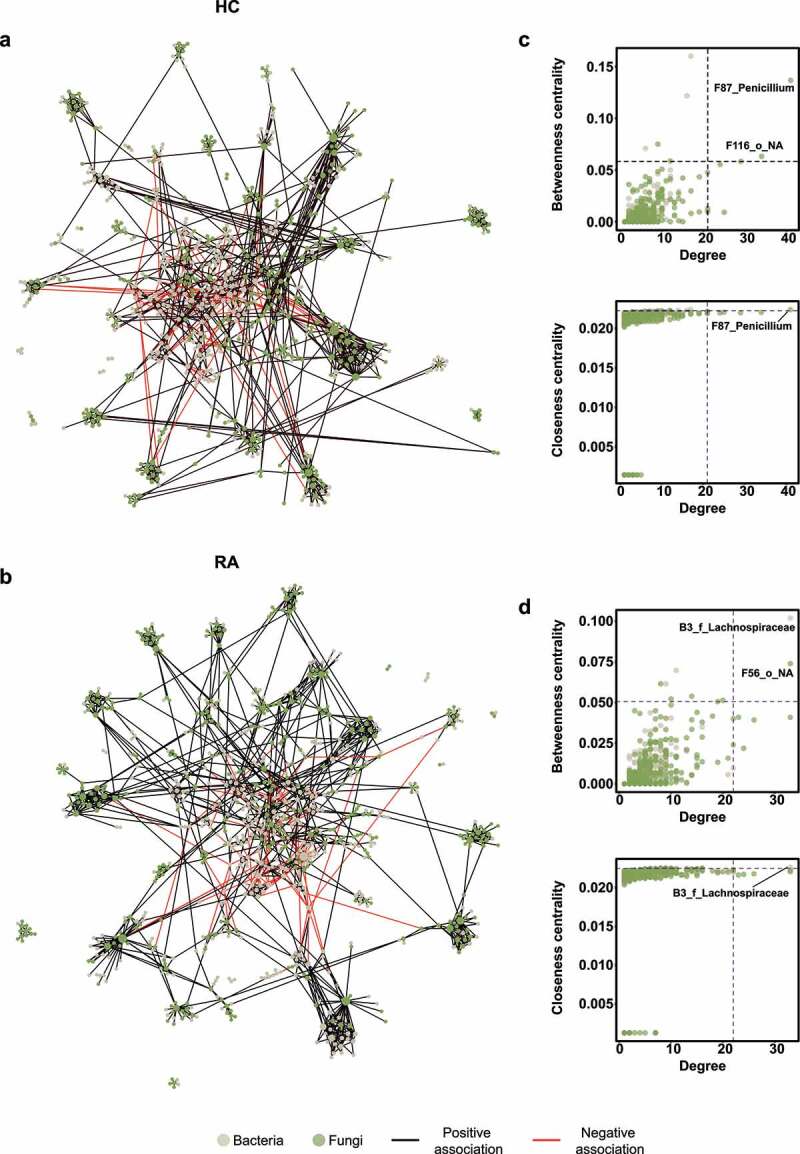
Interkingdom co-occurrence networks and hub nodes of fecal microbiota. (a) Interkingdom HC networks. (b) Interkingdom RA networks. In panels a and b, each node corresponds to an out; edges between nodes correspond to positive (black) or negative (red) correlations inferred from OTU abundance profiles using the SparCC method (*P* < .05, correlation values of < −0.3 or > 0.3). OTUs that belong to different microbial kingdoms are indicated by colors (bacteria, ivory; fungi, green), and node size reflects degree of centrality. (c) Hub nodes of microbial HC networks. (d) Hub nodes of microbial RA networks. In panels c and d, the hub was defined as a node in which degree, betweenness centrality, and closeness centrality were in the top 1%. Dashed lines indicate threshold values of degree, betweenness centrality, and closeness centrality.

We constructed a correlation-based microbial network to investigate microbial associations. The fecal microbial network of the HC group comprised 701 nodes and 1419 edges (Figure 4a), whereas the fecal microbial network of the RA group comprised 801 nodes and 1679 edges (Figure 4b). Degree and betweenness centrality did not significantly differ between the HC and RA groups (Figure S7). There were more fungal nodes in the HC group than in the RA group, and hub composition differed between groups. Hub nodes were defined as nodes in which degree, betweenness centrality, and closeness centrality were in the top 1%. Based on this criterion, the hub of the bacterial–fungal interkingdom network of the HC group was the fungal OTU F87_*Penicillium* (Figure 4c). In the RA group, the hub node was the bacterial OTU B3_f_Lachnospiraceae in the *Lachnospiraceae* family (Figure 4d). These data suggest that fungi influence the microbial community composition in HC, while bacteria associated with dysbiosis influence the microbial community in patients with RA.

### Changes in the fecal fungal community in response to medication

**Figure 5.**
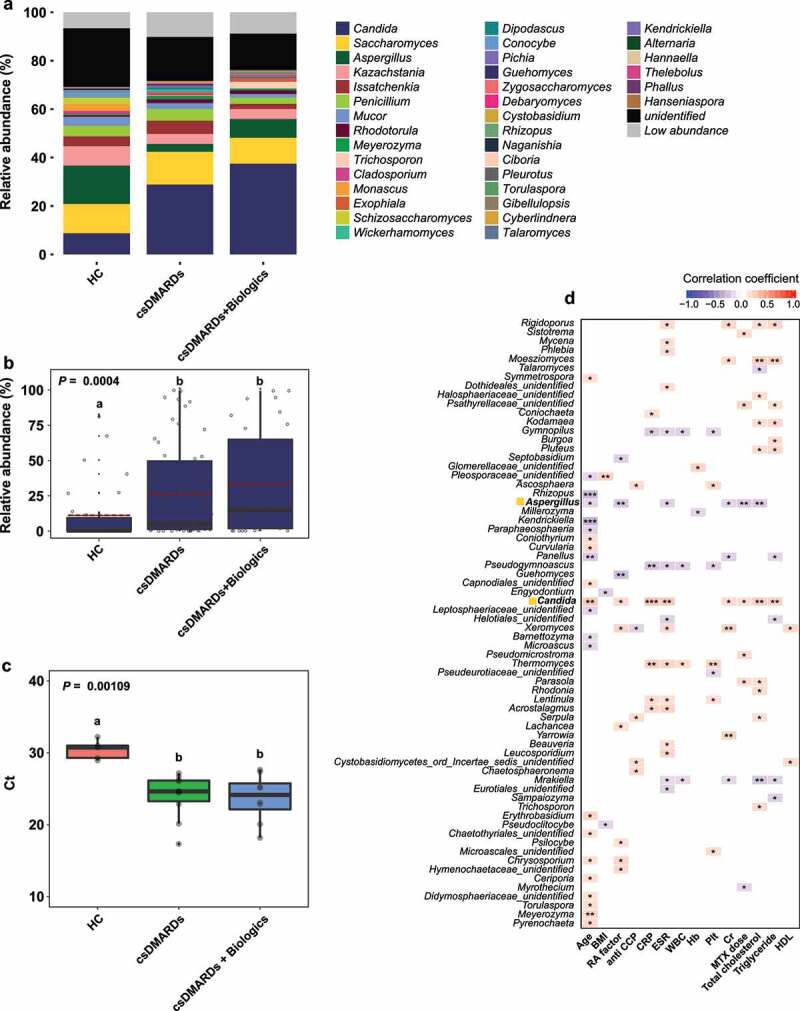
Effects of medication on *Candida* abundance. (a) Fecal microbiota composition according to medication. (b) Relative abundance of *Candida*. Letters indicate statistical significance, as determined by Kruskal–Wallis test followed by Dunn’s test. (c) Results of quantitative PCR analysis of *C. albicans*. Letters indicate statistical significance, as determined by analysis of variance followed by Tukey’s honestly significant difference test. (d) Correlations between relative abundances of fungal OTUs and quantitative variables. Correlation coefficients were estimated using Spearman’s rank correlation. Asterisks indicate statistical significance (***, *P* < .001; **, *P* < .01; *, *P* < .05). Red and blue boxes indicate positive and negative correlations, respectively.

The use of antirheumatic drugs alters the microbial community. For example, etanercept partially alleviated bacterial dysbiosis in patients with RA.^17^ The gut bacterial community can also determine the responses of RA patients to MTX.^23^ We examined the effects of RA therapeutics on the fungal community. We stratified the patients into three groups: csDMARDs (patients treated with csDMARDs; n = 55), csDMARDs + biologics (patients treated with csDMARDs and biologics; n = 32), and biologics (patients treated with biologics; n = 8). Because few patients were treated with biologics, the biologics group was excluded from further analysis. The genus *Candida* was more abundant in the csDMARDs and csDMARDs + biologics groups than in the HC group (Figure 5a, b). However, among patients with RA, the relative abundance of *Candida* was lower in the csDMARDs group than in the csDMARDs + biologics group (Figure 5a). Differences in *Candida* abundance within the RA groups were not statistically significant (Figure 5b). Compared with the csDMARDs + biologics group, the relative abundance of *Aspergillus* was decreased in the csDMARDs group, while the relative abundance of *Penicillium* was increased (Figure 5a).

Polymerase chain reaction (PCR) using *Candida*-specific primers was performed on fecal samples randomly selected from the HC and RA groups. *C. albicans* was a fungal species with significantly greater abundance in RA samples. In the RA group, bands of 200 to 300 bp were observed; such bands were absent in the HC group (Figure S8). The amount of *C. albicans* DNA was significantly greater in the RA group than in the HC group (HC, Ct = 30.4 ± 1.33; RA, Ct = 23.9 ± 3.08). The amount of *C. albicans* DNA was slightly greater in the csDMARDs + biologics group than in the csDMARDs group (csDMARDs, Ct = 24.0 ± 3.02; csDMARDs + biologics, Ct = 23.7 ± 3.19) (Figure 5c).

In terms of clinical factors, the relative abundance of *Candida* was significantly positively correlated with age (Spearman r = 0.29153, *P* = .000802), RF level (Spearman r = 0.20217, *P* = .021579), CRP level (Spearman r = 0.2927, *P* = .000762), ESR (Spearman r = 0.27676, *P* = .000136), MTX dose (Spearman r = 0.18648, *P* = .034349), and total cholesterol level (Spearman r = 0.24822, *P* = .004563) (Figure 5d). The relative abundance of *Aspergillus* was significantly negatively correlated with those factors (RF level: Spearman r = −0.24993, *P* = .004283; ESR: Spearman r = −0.20742, *P* = .018344; MTX dose: Spearman r = −0.22778, *P* = .00943; and total cholesterol level: Spearman r = −0.238, *P* = .006607).

### Fungal dysbiosis can be used for characterization of RA

**Figure 6.**
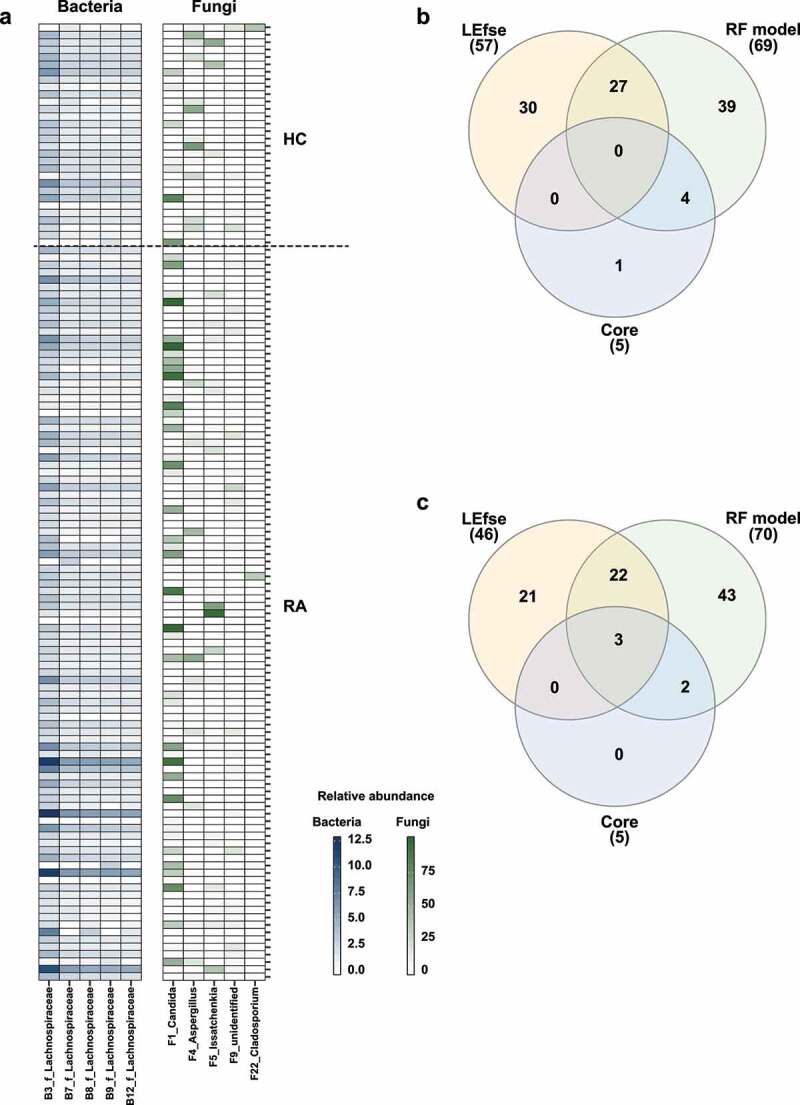
Analysis of core OTUs in fecal microbiota. (a) Core OTUs were identified based on 85% prevalence for bacteria (dark blue) and 70% prevalence for fungi (dark green). Box colors indicate relative abundances of OTUs. Greater color intensity indicates higher relative abundance. Each mark on the y-axis indicates an individual sample. Venn diagrams of the numbers of (b) bacterial and (c) fungal OTUs identified by LEfSe, the random forest model, and core out analysis.

We identified core OTUs (or prevalent OTUs) in fecal samples: core bacterial OTUs were detected in 85% of the 129 fecal samples, while core fungal OTUs were detected in 70% of the fecal samples. Five core bacterial OTUs belonged to *Lachnospiraceae*, whereas five core fungal OTUs belonged to *Candida, Aspergillus, Issatchenkia, Cladosporium*, and an unidentified fungal genus (Figure 6a). Subsequently, three overlapping core fungal OTUs were discovered, but no overlapping core bacterial OTU was identified (Figure 6b, c). Among the bacterial core OTUs, B3_f_Lachnospiraceae, B8_f_Lachnospiraceae, and B9_f_Lachnospiraceae could distinguish between HC and RA groups using a random forest model. However, LEfSe revealed that differences in the relative abundances of these OTUs were not statistically significant (Figures 3, S5). Among the fungal core OTUs, F1_Candida, F4_Aspergillus, and F22_Cladosporium could distinguish between HC and RA groups using both LEfSe and a random forest model (Figures 3, 6c, S6). The association between ACPA and RF, which are serological markers of RA, and the fecal fungal community were investigated. *Aspergillus* and *Candida*, which differed in abundance between the HC and RA groups, were not associated with ACPA. *Aspergillus* was significantly associated negatively with RF, whereas *Candida* was correlated positively (Figure 5d). Therefore, changes in the fungal microbial community, particularly involving *Candida* and *Aspergillus*, could be a feature of RA.

## Discussion

The gut or fecal microbiome plays an important role in several human diseases. We found a distinct fecal microbial composition in patients with RA. Although the relative bacterial abundance differed between the RA and HC groups, alpha diversity did not differ (Figures 1, S1, S3a). CAP and random forest analysis revealed that *Bifidobacterium* and *Streptococcus* were representative of the HC and RA groups, respectively (Figures 2, S5). Similar distributions of bacterial genera in fecal samples have been identified in Asian cohorts.^13,20,24,25^

Fungi affect the composition of the bacterial community.^26–28^ A bacterial–fungal interkingdom network analysis showed that F87_Penicillium was the hub OTU in the HC group, while B3_f_Lachnospiraceae was the hub OTU in the RA group (Figure 4). The *Penicillium* subgenus produces numerous beneficial secondary metabolites, which have antibiotic, antifungal, immunosuppressive, and cholesterol-lowering properties.^29^ The altered relative abundance of *Penicillium* was restored in RA patients via treatment with csDMARDs alone (Figure 5a). *Lachnospiraceae* are reportedly abundant in ACPA-positive patients.^19,30^ Because 75 (76.5%) of our RA patients were ACPA-positive, we hypothesized that the hub OTU shifted from F87_Penicillium to B3_f_Lachnospiraceae in patients with RA. The difference between RA and HC groups was clearer in the fungal community than in the bacterial community. F1_*Candida* and F4_*Aspergillus* were the most differentially abundant fungal genera (Figure 6).

*Aspergillus* caused substantial changes in the fungal community. An OTU that belonged to *Aspergillus* (F4_Aspergillus) was a core fungal OTU (Figure 6a); it was more abundant in the HC group than in the RA group (Figure 3). *Saccharomyces cerevisiae* has a beneficial effect on human health.^31,32^ Alterations in fecal fungal communities have mostly been studied in Western cohorts. We found a significant difference in *Aspergillus* abundance, rather than *Saccharomyces* abundance, between the HC and RA groups. *Saccharomyces* is reportedly more common among individuals who consume a Western diet (e.g., bread, beer, and dairy products), while *Aspergillus* is more common among individuals with a vegetarian diet.^33,34^ In Japan and China, where the diets are similar to the diet consumed in South Korea, *Aspergillus* was more abundant than *Saccharomyces* in the fecal fungal community of healthy adults.^35,36^ Therefore, based on the dietary proportions of vegetables and fermented soybean foods, *Aspergillus* is an essential member of the fecal fungal community in Koreans.^33,34^

Fungi had a substantial effect on fecal microbial community composition in patients with RA; *Candida* was the most abundant fecal genus (Figure 1b, d). *Candida* spp. are frequently detected in the human gastrointestinal tract^33^ and feces;^37^ their abundance is increased in patients with inflammatory bowel disease, cystic fibrosis, and vaginal candidiasis.^38^ We found that medications for RA affected the fungal community composition, such that the abundance of *C. albicans* was enhanced by csDMARDs and biologics (Figure 5c). This is consistent with previous reports of increased *C. albicans* abundance in patients with inflammatory bowel disease (IBD) who were treated with immunosuppressants.^39–41^ During treatment with disease-modifying antirheumatic drugs and tumor necrosis factor-α inhibitors, patients with RA showed an impaired *C. albicans*-specific Th17 response, which led to an increased abundance of *C. albicans*. Although the increased abundances of *C. albicans* in patients with RA and patients with IBD do not exclude the possibility that dysbiosis is caused by disease, they suggest that the dysbiosis is caused by medication. Moreover, although an increased abundance of *C. albicans* may result in opportunistic infections, the risk of candidiasis is low in patients with RA because they retain an effective immune response to *C. albicans*.^42^

The decreased abundance of *Aspergillus* and increased abundance of *Candida* in the feces of patients in our study suggest that such changes are specific to RA. Further studies regarding *Aspergillus* will provide insight into its role in the healthy fecal fungal community and its effect on human health. Our findings suggest that changes in the fungal community could be used as an indicator of fecal dysbiosis in patients with RA.

In conclusion, we investigated dysbiosis and fungal–bacterial interactions in the fecal microbial communities of patients with RA. Changes in fungal communities indicated significant dysbiosis between HC and patients with RA, whereas changes in bacterial communities did not. Future research should examine whether the increased abundance of *C. albicans* is caused by immunosuppressive or immunomodulatory medications. Our results were limited in that they comprised bioinformatics-based predictions of the effects of RA-related changes on fecal microbial communities. *In vivo* experiments are required to confirm that RA alters the fungal community. Therefore, an experimental validation studies concerning the effects of *C. albicans* and *Aspergillus* on RA-related immune pathways are needed. *Aspergillus* was more abundant in the fecal fungal community of healthy Koreans, whereas *Saccharomyces* was comparable to patients with RA. Further research is necessary to clarify precisely our findings differ from the Western cohort.

## Materials and methods

### Sample collection

HC (n = 30) were recruited from the Wonju Severance Christian Hospital. HC who had a chronic, systemic autoimmune disease and pregnant or lactating women were excluded. RA (n = 99), who fulfilled the 2010 ACR/EULAR classification criteria,^43^ were recruited from the Catholic University of Korea Seoul St. Mary’s Hospital. Each individual had been prescribed medication, including non-steroidal anti-inflammatory drugs, corticosteroids, csDMARDs, and biologics. All clinical data were obtained according to established methods, and the DAS28 was used to quantify disease activity.

The Ethics Committees of the Wonju Severance Christian Hospital Ethics Committee (IRB Approval Number: 19–008) and the Catholic University of Korea (IRB Approval Number: KC14TIMI0248) approved this study. Fecal samples were collected from March 2017 to November 2018 and promptly frozen at −20°C. Sequentially collected samples were transported to the laboratory and stored at −80°C before DNA extraction.

### DNA extraction from feces

DNA extraction was performed using the QIAamp PowerFecal Pro DNA Kit (Qiagen, Germany), in accordance with the manufacturer’s instructions. DNA concentration and purity were determined with a Nanodrop 1000 (Thermo Fisher Scientific, USA). The collected DNA was stored at −20°C before amplification by PCR.

### PCR amplification and sequencing

The V3–V4 regions of 16S ribosomal RNA (rRNA) genes were amplified using the Illumina-adapted universal primers 314F/805R. Each PCR reaction contained 12.5 ng of genomic DNA, 2.5 μL of Ex Taq 10× PCR buffer (Takara, Japan), 2.5 μL of dNTP mixture (Takara), 0.125 μL of Takara Ex Taq (Takara), 5 μL of each primer (200 nM final concentration), and distilled water to a total volume of 25 μL. The following thermocycler protocol was used: initial denaturing at 95°C for 3 min; 25 cycles of denaturing at 95°C for 30s, primer annealing at 55°C for 30s, and extension at 72°C for 30s; and final extension at 72°C for 5 min. PCR products were purified using AMPure XP beads (Beckman Coulter, USA), then quantified using a KAPA Library Quantification kit (KAPA Biosystems, USA). Sequencing was conducted on the MiSeq platform using a paired-end 2 ×300 base pairs reagent kit (Illumina, USA).

Subsequently, the fungal internal transcribed spacer 2 (ITS2) region of the 18S ribosomal RNA genes was amplified using the ITS3F/ITS4R primers and i-Starmax II polymerase (Intron Biotechnology, Korea). Each PCR reaction (final volume, 25 μL) contained 2.5 μL of 10× PCR buffer, 2.5 μL of dNTP mixture, 0.31 μL of i-Starmax II polymerase (Intron Biotechnology), 1.25 μL of each primer (500 nM final concentration), and 50–120 ng of genomic DNA. The following thermocycler protocol was used: initial denaturing at 94°C for 4 min; 35 cycles of denaturing at 94°C for 1 min, primer annealing at 60°C for 1 min, and extension at 72°C for 1 min; and final extension at 72°C for 10 min. PCR products were purified using AMPure XP beads (Beckman Coulter). DNA quality and quantity were measured using an Infinite 200 pro (Tecan, Switzerland). All samples were diluted to the same concentration, pooled into a single library, and concentrated using AMPure beads (Beckman Coulter); the pooled library was subjected to gel purification to remove any residual unwanted PCR products. Finally, the pooled library was sequenced on the Illumina MiSeq platform with a read length of 2 × 300 base pairs at the National Environmental Management Center of Seoul National University.

### Sequence processing and filtering

After demultiplexing, overlapping sequences were merged with PEAR, then filtered with the DADA2 plugin^44^ using the “denoise-single” command in QIIME2. Subsequently, high-quality sequences were clustered into OTUs using the open reference vsearch algorithm (vsearch cluster-features-openreference)^45^ against the Silva 99% OTU representative sequence database (version 132, April 2018),^46^ then assembled into an OTU table. Bacterial OTUs were clustered into OTUs using the UCHIME-de novo algorithm,^47^ fungal sequences were checked for chimerism with UCHIME using the June 2017 chimera detection ITS2 database.^48^ Next, the taxonomies of nonchimeric OTUs were assigned using the naïve Bayes algorithm implemented in the q2-feature-classifier, based on the Silva database for the V3–V4 region of the 16S rRNA sequences.^49^ Alternatively, eukaryotes were classified using the UNITE database (UNITE version 7 dynamic of January 2017) for the ITS2 region.^50^

Short bacterial (400 base pairs) and fungal (100–500 base pairs) sequences were used for in-depth analyses. First, OTU tables were imported into R using the readRPM component of the phyloseq package.^51^ Next, sequence data were removed for organisms that had been assigned to non-kingdom-level groups (bacterial OTUs: orders “Chloroplast” and “Rickettsiales;” fungal OTUs: kingdoms “Unassigned,” “Rhizaria,” and “Metazoa”). Subsequently, false positive OTUs were removed from stool samples, while singleton OTUs were eliminated from all samples. This process reduced the total bacterial OTU count from 1346 to 1338 and total fungal OTU count from 1641 to 1595. The remaining 1338 bacterial OTUs and 1595 fungal OTUs were used for further analysis.

### Statistical analyses and visualization

Statistical analysis was performed using R statistical software, version 3.5.2.^52^ After multiple hypothesis tests had been corrected using the false discovery rate method, significant results were determined using a *p*-value threshold of 0.05. First, OTU tables were scaled by cumulative-sum scaling (CSS) and log-transformed (for normalization) using the *cumNum* and *MRcounts* functions in the metagenomeSeq package in R.^53^ Next, rarefication of bacterial (5425 reads) and fungal (4256 reads) reads was conducted using the *rarefy_even_depth* function in the Phyloseq package in R; this was followed by calculation of the Shannon and Simpson indices using the *diversity* function in the Vegan (version 2.5–3) package in R. The Wilcoxon rank-sum test and one-way analysis of variance were also used. A Bray–Curtis dissimilarity matrix was produced for use in two separate principal coordinates analyses; CAP was then performed using RA and HC constraints, respectively, with the *capscale* and *ordinate* functions from the Vegan and Phyloseq packages. PERMANOVA using the *adonis* function in the Vegan package (version 2.5–3) was also used for analysis.^54^ Subsequently, the core OTUs of RA and HC groups were identified using a prevalence threshold of 85% for bacteria and 70% for fungi. Differentially abundant OTUs between the RA and HC groups were identified using LEfSe (https://huttenhower.sph.harvard.edu/galaxy/).^55^ Differences in OTU abundance were considered significant when *p*-values were < 0.05.

### Microbial correlation networks

Bacterial–fungal networks were constructed to infer hub and complex OTU associations for RA and HC groups. Because the number of participants differed between the RA (n = 99) and HC (n = 30) groups, 30 samples from the RA group were randomly subsampled using the sample function in R software to avoid differences in network properties based on differences in sample size. Thus, we obtained 1302 OTUs for the HC group and 1428 OTUs for the RA group; these OTUs were used to construct interkingdom co-occurrence networks. In contrast, CSS-normalized OTU abundance tables that included bacteria and fungi were used as an input for SparCC;^56^ significant associations between OTUs were restricted to OTUs with correlations of > 0.3 and < −0.3 (*P* < .05).^57^ Co-occurrence networks were visualized with Gephi (version 0.9.2)^58^ using the ForceAtlas2 layout. Within the networks, the proportions of inter-kingdom (associations between bacteria and fungi) and intra-kingdom (associations within the same kingdom) links were quantified and displayed in bar graph format.^59^ Specifically, HC and RA networks were compared in terms of degree, betweenness centrality, closeness centrality, and eigenvector centrality; these values were computed using igraph (version 1.2.1).^60^ The hub OTUs of each network were defined as the top 1% of OTUs in terms of degree, betweenness centrality, and closeness centrality. For the RA group, OTUs of degree > 20, betweenness centrality > 0.05343148, and closeness centrality > 0.01229823 were defined as hub OTUs. For the HC group, OTUs of degree > 19.7, betweenness centrality > 0.06493721, and closeness centrality > 0.02258181 were identified as hub OTUs.

### Fungal strain cultivation

*C. albicans* (KCCM 11282) was obtained from the Korean Culture Center of Microorganisms (Korea). *C. albicans* was cultivated and maintained in yeast extract peptone dextrose (YPD) agar plates or YPD broth at 25°C. Viable cell numbers were determined by spreading serially diluted culture medium on YPD agar plates. Absorbance at 550 nm was measured using a microplate reader (Tecan).

### DNA extraction and qualitative PCR (gel blotting of samples)

Single *C. albicans* colonies were inoculated and cultivated in YPD broth for 24 h. Broth cultures were centrifuged at 12,000 g for 5 min; DNA was then extracted from cell pellets using the QIAamp PowerFecal Pro DNA Kit (Qiagen). Next, genomic DNA was amplified using i-Starmax II polymerase (Intron Biotechnology). Each PCR reaction (final volume, 25 μL) contained 2.5 μL of 10× PCR buffer, 2.5 μL of dNTP mixture, 0.31 μL of i-Starmax II polymerase (Intron Biotechnology), 1.25 μL of each primer (500 nM final concentration), and 50–120 ng of genomic DNA. The following thermocycler protocol was used: initial denaturation at 94°C for 4 min; 35 cycles of denaturation at 94°C for 1 min, primer annealing at 60°C for 1 min, and extension at 72°C for 1 min; and final extension at 72°C for 10 min. The following primers were used: forward, 5′-TTTATCAACTTGTCACACCAGA-3′; reverse, 5′-ATCCCGCCTTACCACTACCG-3′.^61^ PCR products were separated by electrophoresis on a 1.5% agarose gel containing SYBR DNA SafeStain (Thermo Fisher Scientific, USA). Bands were visualized using a ChemiDoc device (Thermo Fisher Scientific).

### Real-time quantitative PCR to quantify C. albicans abundance

Real-time quantitative PCR was conducted with a QuantStudio 6 Flex Real-Time PCR system (Applied Biosystems, USA) using PowerUp™ SYBR® Green Master Mix (Thermo Fisher Scientific). The primers used for qualitative PCR of *C. albicans* were used for real-time quantitative PCR. Each PCR reaction (final volume, 20 μL) contained 10 μL of SYBR Green Master Mix, 0.8 μL of each primer, and 2 μL of genomic DNA. The following thermocycler protocol was used: denaturation at 95°C for 2 min, followed by 40 cycles of 95°C for 15s and annealing at 60°C for 60s. Amplification specificity was evaluated by melt curve analysis.

## Supplementary Material

Supplemental Material

## Data Availability

All raw sequences derived from this experiment were submitted into the Sequence Read Archive of NCBI and can be found under the BioProject accession number PRJNA791216. Metadata files, R data files, and R notebooks for full analyses are available from https://github.com/hyunkim90/rheumatoid_arthritis_and_mycobiota

## List of abbreviations

ACPA: Anti-citrullinated protein antibody
CAP: Canonical analysis of principal coordinates
CRP: C-reactive protein
CSS: Cumulative-sum scaling
csDMRADs: Conventional synthetic disease-modifying antirheumatic drugs
ESR: Erythrocyte sedimentation rate
HC: Healthy controls
IBD: Inflammatory bowel disease
LEfSe: Linear discriminant analysis effect size
OTU: Operational taxonomic units
PCoA: Principal coordinate analysis
PCR: Polymerase chain reaction
PERMANOVA: Permutational analysis of variance
RA: Rheumatoid arthritis
RF: Rheumatoid factor
rRNA: ribosomal RNA
YPD: Yeast extract peptone dextrose
