# Supplementary material for: Dysbiotic but nonpathogenic shift in the fecal mycobiota of patients with rheumatoid arthritis
Source: Gut Microbes. 2022 Dec 6;14(1):2149020. doi: 10.1080/19490976.2022.2149020 (PMC9728469; doi:10.1080/19490976.2022.2149020)
Supplement: Supplemental Material [file KGMI_A_2149020_SM2397.docx]

**Supplementary file for**

**Dysbiotic but nonpathogenic shift in the fecal mycobiota in patients with rheumatoid arthritis**

Eun Ha Lee^ab†^, Hyun Kim^c†^, Jung Hee Koh^de†^, Kwang Hyun Cha^a^, Kiseok Keith Lee^c‡^, Wan-Uk Kim^de*^, Cheol-Ho Pan^a*^, and Yong-Hwan Lee^bcfgh*^

^a^Natural Product Informatics Research Center, KIST Gangneung Institute of Natural Products, Gangneung, Korea; ^b^Interdisciplinary Program in Agricultural Genomics, Seoul National University, Seoul, Korea; ^c^Department of Agricultural Biotechnology, Seoul National University, Seoul, Korea; ^d^Division of Rheumatology, Department of Internal Medicine, College of Medicine, the Catholic University of Korea, Seoul, Korea; ^e^Center for Integrative Rheumatoid Transcriptomics and Dynamics, College of Medicine, the Catholic University of Korea, Seoul, Republic of Korea; ^f^Center for Plant Microbiome Research, Seoul National University, Seoul, Korea; ^g^Plant Immunity Research Center, Seoul National University, Seoul, Korea; ^h^Research Institute of Agriculture and Life Sciences, Seoul National University, Seoul, Korea.

***Corresponding authors**

Wan-Uk Kim, [wan725@catholic.ac.kr](mailto:wan725@catholic.ac.kr); Cheol-Ho Pan, [panc@kist.re.kr](mailto:panc@kist.re.kr); Yong-Hwan Lee, [yonglee@snu.ac.kr](mailto:yonglee@snu.ac.kr)

^†^ These authors contributed equally: Eun Ha Lee; Hyun Kim; Jung Hee Koh

^‡^Present address: Department of Ecology and Evolution, The University of Chicago, 1101 East 57th Street, Chicago, IL, 60637 USA

**This file contains Figures S1 to S8 and Tables S1 to S2.**

**Figure S1**


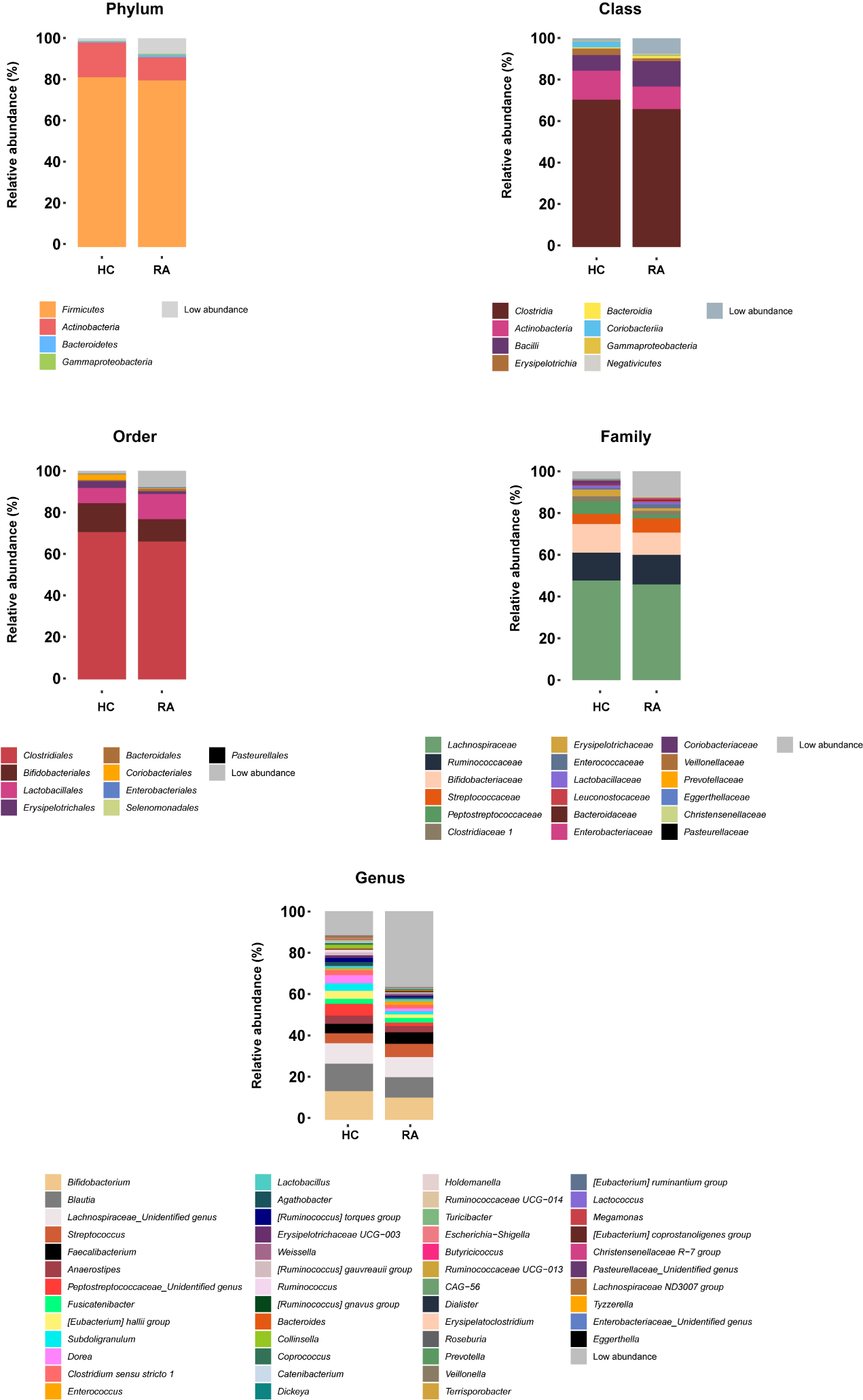


**Figure S1. Comparisons of the fecal bacterial community composition between HC and RA.** Community composition was compared at the phylum, class, order, family, and genus levels, respectively. The columns of different colors represent different taxa, and the height of the bars represents the proportions of each taxon. Genera with abundance <0.3% are grouped as "Low abundance." HC, healthy controls; RA, patients with RA.

**Figure S2**

**
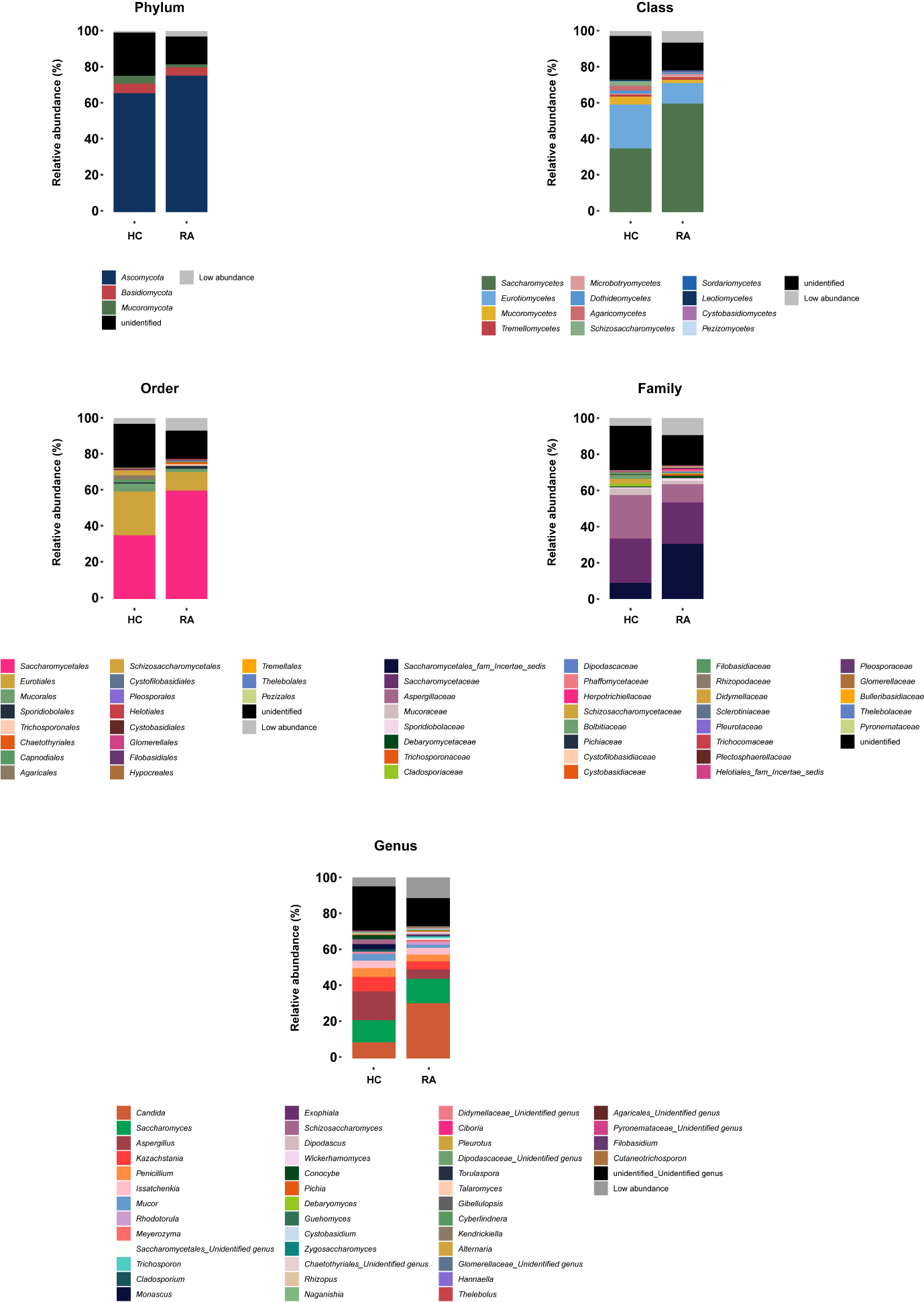
**

**Figure S2. Comparisons of the fecal fungal community composition between HC and RA.** Community composition was compared at the phylum, class, order, family, and genus levels, respectively. The columns of different colors represent different taxa, and the height of the bars represents the proportions of each taxon. Genera with abundance <0.3% are grouped as "Low abundance." HC, healthy controls; RA, patients with RA.

**Figure S3**

**
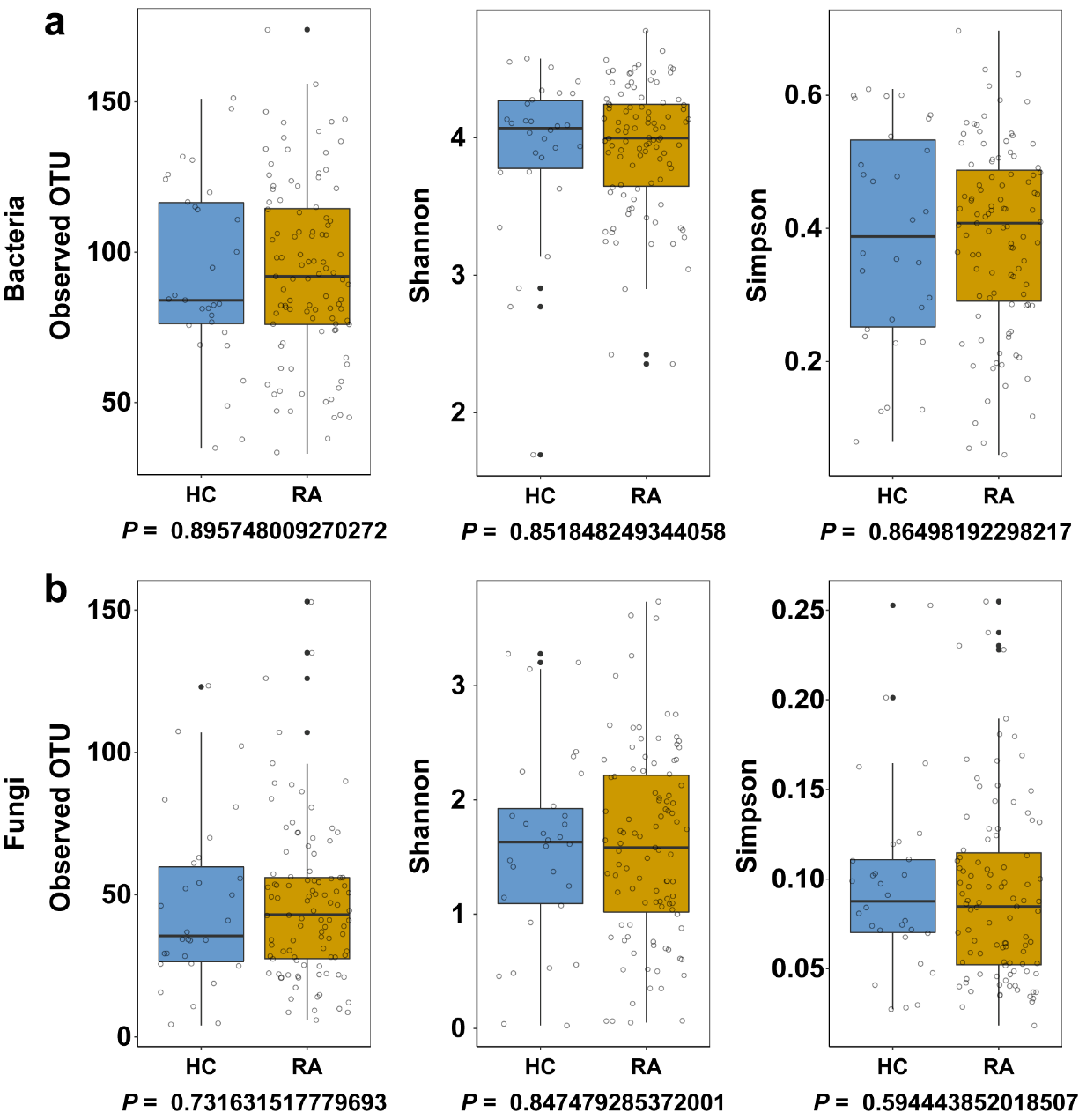
**

**Figure S3. Differences in alpha diversity between HC and RA.** (a) Alpha diversity metrics for the fecal bacterial community**.** (b) Alpha diversity metrics for the fecal fungal community. Boxes and lines in the boxes represent the inter-quantile range (Q3–Q1) and median of diversity values, respectively. Black-filled dots indicate potential outliers. Lower and upper whiskers show minimum and maximum alpha diversity values in each group. The gray dots correspond to the exact values of the diversity indices of each sample. Statistical significance was estimated using a two-sided Mann–Whitney test. HC, healthy controls; RA, patients with RA.

**Figure S4**

**
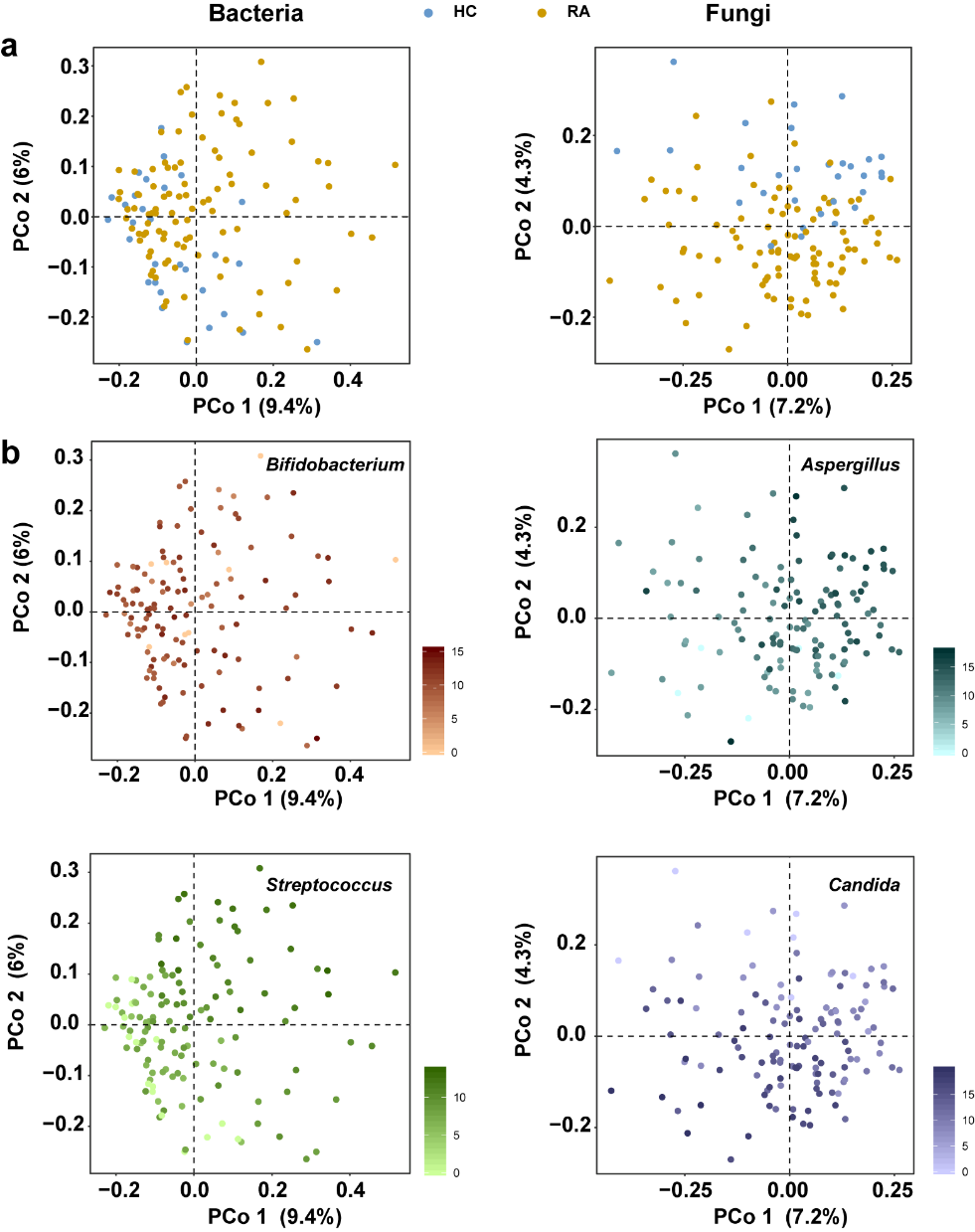
**

**Figure S4. Unconstrained principal coordinate analysis (PCoA) of bacterial and fungal communities between HC and RA.** (a) The data ordination from the beta diversity metrics for human fecal microbial community structure**.** (b) Ordination analysis indexed based on abundant genera of bacterial (left) and fungal (right) communities. HC, healthy controls; RA, patients with RA.

**Figure S5**


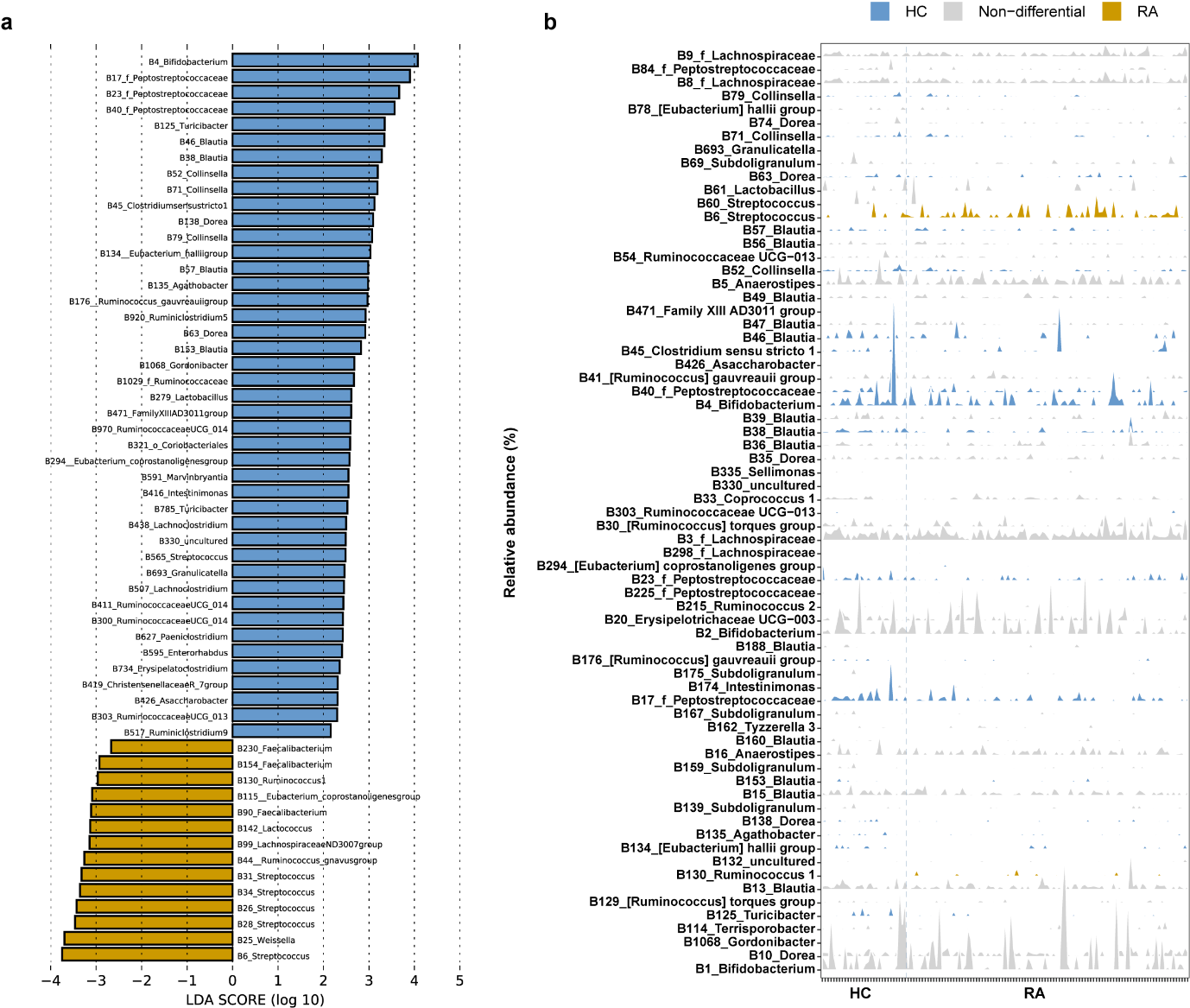


**Figure S5. Fecal bacterial OTUs affected by the dysbiosis of rheumatoid arthritis.** (a) Differentially abundant bacterial OTUs estimated from the LEfse analysis between HC and RA. Blue and yellow bars indicate enrichment of OTUs in HC and RA, respectively. The size of the bars corresponds to the logarithmic discriminant analysis (LDA) score. The threshold of LDA score is 2**.** (b) Bacterial OTUs discriminating between the compositional differences in HC and RA using a random forest classification model. OTUs are colored based on their categorization as “HC-enriched” and “RA-enriched” groups based on their differential abundance test results. Each tick on the x-axis indicates an individual control HC and RA sample. HC, healthy controls; RA, patients with RA.

**Figure S6**

**
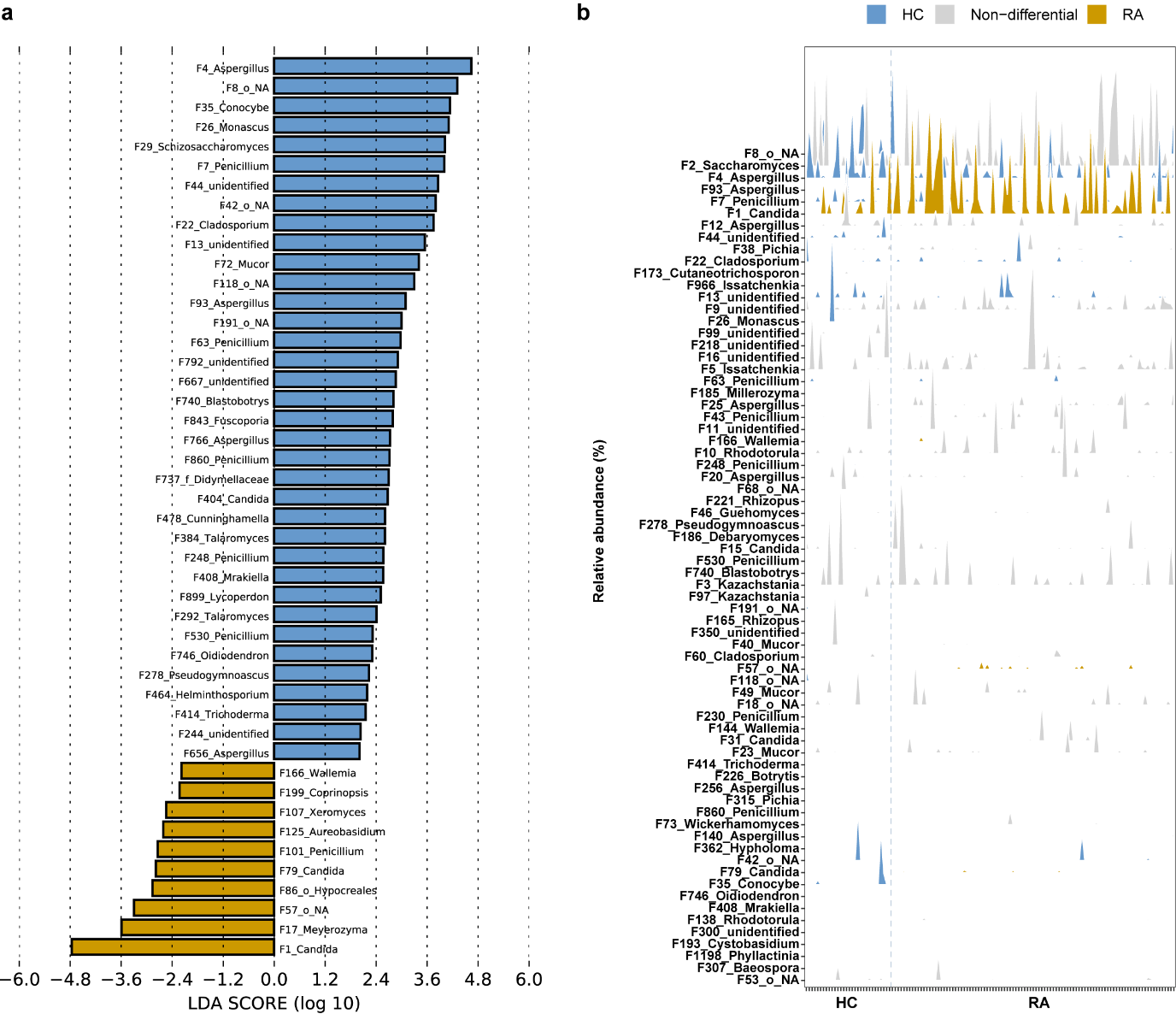
**

**Figure S6. Fecal fungal OTUs affected by the dysbiosis of rheumatoid arthritis.** (a) Differentially abundant fungal OTUs estimated from the LEfse analysis between HC and RA. Blue and yellow bars indicate the enrichment of OTUs in HC and RA, respectively. The size of the bars corresponds to the logarithmic discriminant analysis (LDA) score. The threshold of LDA score is 2**.** (b) Fungal OTUs discriminating between the compositional differences in HC and RA using a random forest classification model. OTUs are colored based on their categorization as “HC-enriched” and “RA-enriched” groups according to the results of the differential abundance test. Each tick on the x-axis indicates an individual control HC and RA sample. HC, healthy controls; RA, patients with RA.

**Figure S7**

**
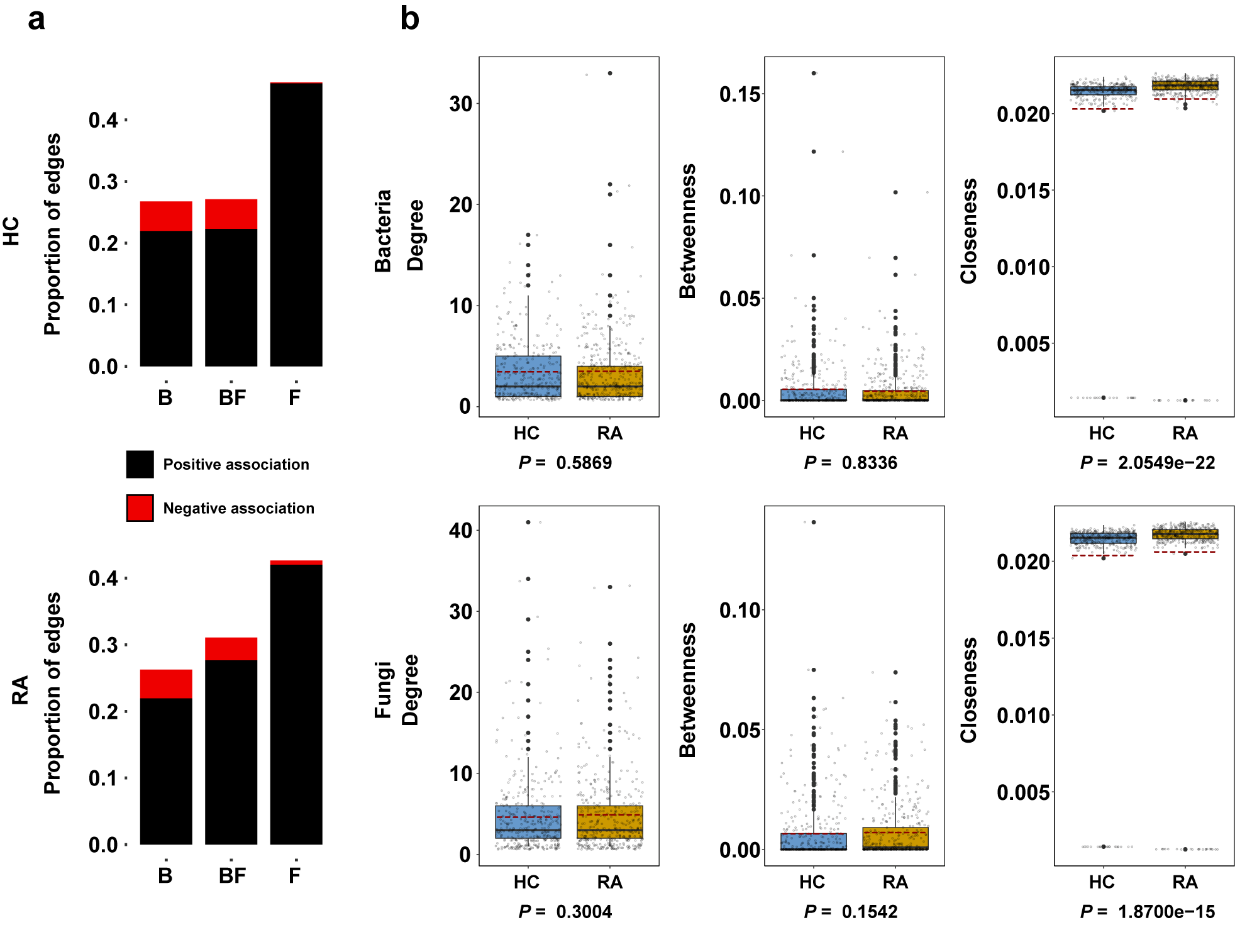
**

**Figure S7. A comparison of the topological properties between microbial HC and RA networks.** (a) The proportion of edges (associations) comprising microbial Healthy (upper panel) and RA (bottom panel) networks. The proportion of positive and negative associations is indicated as black- and red-colored bars, respectively. B, bacterial–bacterial association; BF, bacterial–fungal association, F, fungal–fungal association. (b) Comparison of the topological properties of microbial control and RA networks. Pairwise comparison of the topological properties of bacterial nodes is indicated on the upper panel, whereas that of fungal nodes is displayed on the bottom panel. HC, healthy controls; RA, patients with RA.

**Figure S8**

**
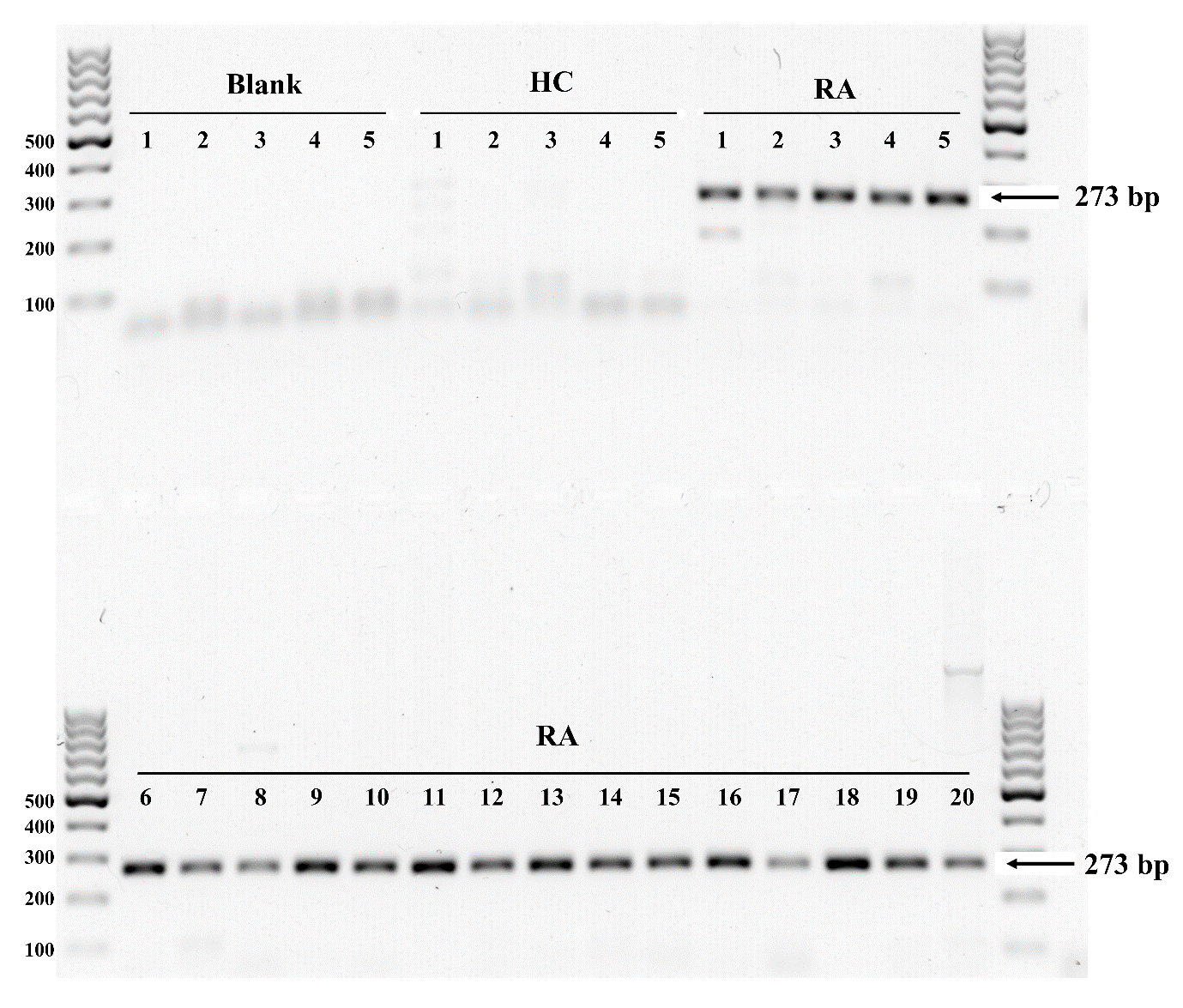
**

**Figure S8. Agarose gel electrophoresis image of the *C. albicans* specific PCR products.** Control and HC exhibit weak primer bands at the bottom. RA confirmed the presence of *C. albicans* by observing significant bands at 273 bp. Blank, water; HC, healthy controls; RA, patients with RA.

**Table S1. The medication for patients with RA.**

| **Medication, n (%)** | **Healthy controls**  **(*n* = 30)** | **Patients with RA**  **(*n* = 99)** |
| --- | --- | --- |
| **csDMARDs** |  |  |
| MTX | - | 56 (56.6 %) |
| Leflunomide | - | 42 (42.4 %) |
| Hydroxychloroquine | - | 40 (40.4 %) |
| Sulfasalazine | - | 15 (15.2 %) |
| **Biologics** |  |  |
| Eetanercept | - | 3 (3.0 %) |
| Adalimumab | - | 3 (3.0 %) |
| Abatacept | - | 17 (17.2 %) |
| Tocilizumab | - | 17 (17.2 %) |
| **Others** |  |  |
| Tofacitinib | - | 1 (1.0 %) |
| Tacrolimus | - | 13 (13.1 %) |
| Glucocorticoids | - | 69 (69.7 %) |
| NSAIDs | - | 54 (54.5 %) |

**Table S2. Results on permutational analysis of variance (PERMANOVA)**

| **Bacteria** | Df | SumsOfSqs | MeanSqs | F.Model | R^2^ | Pr(>F) |  |
| --- | --- | --- | --- | --- | --- | --- | --- |
| Diagnosis | 1 | 0.568 | 0.56787 | 2.2563 | 0.01746 | 2.00E-04 | *** |
| Residuals | 127 | 31.963 | 0.25168 |  | 0.98254 |  |  |
| Total | 128 | 32.531 |  |  | 1 |  |  |
|  | Df | SumsOfSqs | MeanSqs | F.Model | R2 | Pr(>F) |  |
| Age | 1 | 0.466 | 0.46553 | 1.86272 | 0.01431 | 0.002 | ** |
| BMI | 1 | 0.343 | 0.34328 | 1.37359 | 0.01055 | 0.0493 | * |
| Total_cholesterol | 1 | 0.333 | 0.33307 | 1.33274 | 0.01024 | 0.0705 | . |
| Duration | 2 | 0.574 | 0.28697 | 1.14825 | 0.01764 | 0.1493 |  |
| HDL | 1 | 0.203 | 0.20323 | 0.81318 | 0.00625 | 0.8217 |  |
| Triglyceride | 1 | 0.353 | 0.35321 | 1.41331 | 0.01086 | 0.0412 | * |
| RA_factor | 1 | 0.167 | 0.16688 | 0.66775 | 0.00513 | 0.9732 |  |
| anti_CCP | 1 | 0.269 | 0.26944 | 1.07813 | 0.00828 | 0.3117 |  |
| CRP | 1 | 0.35 | 0.35031 | 1.40172 | 0.01077 | 0.0454 | * |
| ESR | 1 | 0.232 | 0.23171 | 0.92715 | 0.00712 | 0.5988 |  |
| Residuals | 117 | 29.24 | 0.24992 |  | 0.89885 |  |  |
| Total | 128 | 32.531 |  |  | 1 |  |  |
| --- |  |  |  |  |  |  |  |
| **Fungi** | Df | SumsOfSqs | MeanSqs | F.Model | R^2^ | Pr(>F) |  |
| Diagnosis | 1 | 0.885 | 0.88533 | 2.8042 | 0.0216 | 1.00E-04 | *** |
| Residuals | 127 | 40.096 | 0.31572 |  | 0.9784 |  |  |
| Total | 128 | 40.981 |  |  | 1 |  |  |
|  | Df | SumsOfSqs | MeanSqs | F.Model | R2 | Pr(>F) |  |
| Age | 1 | 0.583 | 0.58312 | 1.84445 | 0.01423 | 0.0002 | *** |
| BMI | 1 | 0.26 | 0.26037 | 0.82356 | 0.00635 | 0.8672 |  |
| Total_cholesterol | 1 | 0.504 | 0.50378 | 1.5935 | 0.01229 | 0.0032 | ** |
| Duration | 2 | 0.758 | 0.37907 | 1.19903 | 0.0185 | 0.0546 | . |
| HDL | 1 | 0.319 | 0.31886 | 1.00858 | 0.00778 | 0.4489 |  |
| Triglyceride | 1 | 0.272 | 0.2715 | 0.85879 | 0.00663 | 0.8059 |  |
| RA_factor | 1 | 0.383 | 0.38336 | 1.21261 | 0.00935 | 0.1014 |  |
| anti_CCP | 1 | 0.304 | 0.30386 | 0.96113 | 0.00741 | 0.56 |  |
| CRP | 1 | 0.338 | 0.33806 | 1.06932 | 0.00825 | 0.3041 |  |
| ESR | 1 | 0.271 | 0.27083 | 0.85665 | 0.00661 | 0.8056 |  |
| Residuals | 117 | 36.989 | 0.31615 |  | 0.90259 |  |  |
| Total | 128 | 40.981 |  |  | 1 |  |  |
| Signif. codes: 0 ‘***’ 0.001 ‘**’ 0.01 ‘*’ 0.05 ‘.’ 0.1 ‘ ’ 1 | | | | | | | |
